# The performance of rapid plasma reagin (RPR) titer in HIV-negative general paresis after neurosyphilis therapy

**DOI:** 10.1186/s12879-018-3062-4

**Published:** 2018-04-02

**Authors:** Ying Jiang, Ruihui Weng, Yuefeng Zhang, Rong Fan, Yulun Liu, Zhigang Chen, Fuhua Peng, Yong Chen, Xiaohong Chen

**Affiliations:** 1 Department of Neurology, The Third Affiliated Hospital, Sun Yat-sen University, 600 Tianhe Road, Guangzhou, Guangdong 510630 People’s Republic of China; 2 Department of Neurology, Guangzhou Brain Hospital, Affiliated Brain Hospital of Guangzhou Medical University, 36 Mingxin Road, Guangzhou, Guangdong 510370 People’s Republic of China; 3 Department of Biostatistics, Epidemiology and Informatics, University of Pennsylvania, Philadelphia, PA 19104 USA

**Keywords:** Neurosyphilis, General paresis, Rapid plasma regain, HIV-negative

## Abstract

**Background:**

Repeated nontreponemal serologic test for syphilis titers is recommended to evaluate treatment response. However, it is unknown whether serum rapid plasma reagin (RPR) titer can serve as a surrogate for determining the efficacy of treatment in general paresis (GP) remains unknown.

**Methods:**

We retrospectively reviewed data from 105 GP patients, who were divided into two groups (62 CSF RPR+ patients and 43 CSF RPR- patients) according to reactive RPR test status in CSF. Clinical assessment included the Mini-Mental State Examination (MMSE) scores, CSF examinations (WBC count, protein concentration and RPR titer), and serum tests (RPR titer and TPPA). Among the 105 GP patients, 13 CSF RPR+ patients and 6 CSF RPR- patients had a 12 months follow-up of CSF, serum measures and MMSE.

**Results:**

The median serum RPR titer was significantly higher in CSF RPR+ patients than that in CSF RPR- GP patients, 1:8 [IQR 1:4–1:32] vs. 1:4 [IQR 1:4–1:8] (*P* < 0.001). The number of CSF RPR+ patients with serum RPR titer≥1:32 was significantly higher when compared with CSF RPR- patients (*P* = 0.001). For CSF RPR+ patients, the MMSE scores improved or remained constantly after penicillin treatment. For CSF RPR+ patients, the CSF RPR titer declined four-fold in 85% (11/13) of the patients, whereas the serum RPR titer declined four-fold in only 46% (6/13) of the patients, the odds ratio is 6.4 (95% confidence interval 1.0–41.2).

**Conclusions:**

A four-fold decline in CSF RPR titer is a good predictor for treatment efficacy in CSF RPR+ GP patients within 12 months after the completion of therapy.

**Electronic supplementary material:**

The online version of this article (10.1186/s12879-018-3062-4) contains supplementary material, which is available to authorized users.

## Background

Syphilis, which is caused by *Treponema pallidum*, is a major health problem worldwide. Cases of syphilitic dementia (general paresis, GP), which is characterized by rapidly progressive cognitive decline with or without psychiatric features, continue to be reported in the modern era [1].

Serum treponemal and non-treponemal tests are the most widely used methods for syphilis diagnosis. Reactive serum treponemal test (TT, including *T. pallidum* Haemagglutination test (TPHA) and *T. pallidum* Particle Agglutination test (TPPA)), symptoms and signs of neurosyphilis and reactive venereal disease research laboratory (VDRL) in cerebrospinal fluid (CSF) or CSF white blood cells (WBCs) > 5/μl or CSF protein > 45 mg/dL are used in the diagnosis of symptomatic neurosyphilis [2]. A reactive rapid plasma reagin (RPR) test in CSF is considered to be the diagnostic of neurosyphilis [2, 3], but this test may be nonreactive in patients with GP depending on the criteria used to define neurosyphilis.

Repeated titers of nontreponemal (i.e., RPR, VDRL) serologic test for syphilis has been recommended to evaluate treatment response, with a four-fold decrease from baseline and/or seroreversion in 12–24 months after treatment representing an appropriate response to therapy/serologic cure [4]. For those neurosyphilis with symptoms or signs, resolution of clinical abnormalities is also considered to determine the efficacy of treatment [5].

The goal of this study is to explore whether serum RPR titer could serve as a surrogate for determining the efficacy of treatment in GP.

## Methods

### Patients

We retrospectively reviewed 105 GP patients (91 males and 14 females). These GP patients were late syphilis and fulfilled all of the following criteria: (1) TPPA and RPR titer were tested in serum and CSF, and were positive in serum TPPA; (2) serologic test was based on RPR positivity in CSF, or WBC count of CSF>5 cells/μL or protein of CSF>45 mg/dL with TPPA positivity in CSF [2]; (3) patient has slowly progressive deterioration in memory, personality, and habits, and with disorientation; (4) exclusion of other diagnosis. These patients were divided into two groups (62 CSF RPR+ patients and 43 CSF RPR- patients) according to reactive RPR status in CSF. Furthermore, since some GP patients were uncooperative, certain information was obtained from their relatives at admission. All patients involved in this study were diagnosed with GP for the first time and they have not received the recommended penicillin therapy [penicillin 18–24 million units intra-venous (IV) daily for 10–14 days] before the diagnosis of GP. Cognitive assessment, the Chinese version of the Mini-Mental State Examination (MMSE), was tested in all cases. It covers the following five areas of cognition: orientation, memory, attention and calculation, recall and language [6]. WBC count, protein concentration and RPR titer in CSF, RPR titer and TPPA in serum were assessed. Human immunodeficiency virus (HIV) infection was excluded in all patients using serological testing.

Thirteen CSF RPR+ GP patients returned for follow-up visits with lumbar puncture, MMSE scores and blood samples in 12 months after penicillin treatment (penicillin G, 24 million units per day for 14 days) (see Additional file 1: Table S1). Fourteen CSF RPR- GP patients returned for follow-up visits with MMSE scores and serological tests in 12 months after penicillin treatment, while 6 of them had a follow-up evaluation of CSF (see Additional file 2: Table S2). An appropriate response to the therapy in the follow-up visit was defined as a four-fold decline in RPR titer which means four-fold decrease or returning to non-reactive in 12 months after treatment.

### Statistical analysis

All statistical analyses were performed by the Statistical Program for Social Sciences (SPSS) software (version 22.0, Chicago, IL, USA). Associations between categorical variables were assessed using the χ2 test or Fisher’s exact test. Associations between continuous variables and categorical variables were assessed using the Mann-Whitney *U* test. *P* < 0.05 was considered to be statistically significant.

### Ethics

The study was conducted according to the principles expressed in the Declaration of Helsinki and approved by the Medical Ethics Committee of the Third Affiliated Hospital of Sun Yat-sen University. The patients or the guardians of patients with severe cognitive impairment provided written informed consent. Lumbar puncture was also performed with informed consent.

## Results

### Patients

**Fig. 1:**
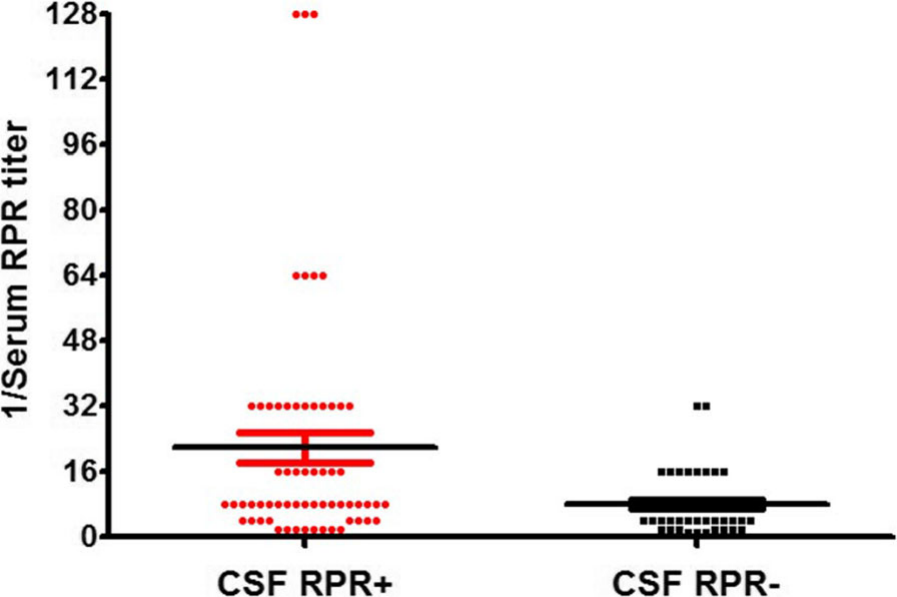
The serum RPR titers for CSF RPR+ and CSF RPR- patients in a scatter plot

One hundred and five GP patients were divided into two groups (62 CSF RPR+ patients and 43 CSF RPR- patients) according to reactive RPR test status in CSF. The characteristics and CSF abnormalities of GP patients with CSF RPR+ and CSF RPR- are shown in Tables 1 and 2. The serum RPR titers of CSF RPR+ and CSF RPR- patients are shown in Fig. 1. The median serum RPR titer was significantly higher in CSF RPR+ patients than that in CSF RPR- GP patients, 1:8 [IQR 1:4–1:32] vs. 1:4 [IQR 1:4–1:8] (*P* < 0.001, see Table 1). The number of CSF RPR+ patients with serum RPR titer≥1:32 was significantly higher when compared with CSF RPR- patients (*P* = 0.001). The number of CSF RPR+ GP patients who had no abnormalities or only one abnormality on initial lumbar puncture was significantly different when compared with CSF RPR- patients (see Table 2). Furthermore, no significant correlation was found between the 1/CSF RPR titer and 1/serum RPR titer in CSF RPR+ GP patients. (*r* = 0.149, *P* = 0.249).

**Table 1:** Characteristics, CSF and serum measures in CSF RPR+ and CSF RPR- patients

Characteristic	All Patients (*n* = 105)	CSF RPR+ Patients (*n* = 62)	CSF RPR- Patients (*n* = 43)	*P* ^a^
Male sex	91/105(87%)	54/62(87%)	37/43(86%)	0.876
Age, median years (IQR)	52 (47–58)	53(47–58)	52 (47–56)	0.432
MMSE (IQR)	14(12–17)	14(12–17)	14(11–17)	0.982
CSF WBC count				
> 5 cells/μl	26/89(29%)	14/46(30%)^b^	12/43(28%)	0.793
Median cells/ml (IQR)	3(1–6)	3(0.75–6)	2(1–6)	0.878
CSF protein concentration				
> 45 mg/dl	73/89(82%)	34/46(74%)^b^	39/43(91%)	0.039
Median mg/dl (IQR)	62(48–75)	62.5(45–75.25)	61(50–75)	0.577
Reactive CSF RPR	62/105 (59%)	62/62(100%)	0/43(0%)	
Median CSF RPR titer (IQR)	–	1:2 (1:2–1:4)	–	
Serum RPR titer				
≥ 1: 32	21/108(19%)	19/62(31%)	2/43(4.7%)	0.001
1: 16	16/108(15%)	8/62(13%)	8/43(18.6%)	0.424
1: 1–1: 8	68/108(63%)	35/62(56%)	33/43(76.7%)	0.032
Median serum RPR titer (IQR)	1:8(1:4–1:16)	1:8(1:4–1:32)	1:4(1:4–1:8)	< 0.001
Normalized by final follow-up visit	14/27(52%)	6/13(46%)^c^	8/14(57%)^d^	0.568

Data are no. (%) of patients, unless otherwise indicated. *IQR* interquartile range, *RPR* rapid plasma regain, *MMSE* mini-mental state examination

^a^Associations between categorical variables were assessed using the ×2 test or Fisher’s exact test. Associations between continuous variables and categorical variables were assessed using the Mann-Whitney U test

^b^Data are for 46 patients

^c^Data are for 13 patients

^d^Data are for 14 patients

**Table 2:** Cerebrospinal fluid abnormalities in CSF RPR+ and CSF RPR- patients

Characteristic	All Patients (*n* = 89)	CSF RPR+ Patients (*n* = 46)	CSF RPR- Patients (*n* = 43)	*P*
No abnormalities	8/89(9%)	8/46(17%)	0/43(0)	0.004
One abnormality	63/89(71%)	28/46(61%)	35/43(81%)	0.033
Two abnormalities	18/89(20%)	10/46(22%)	8/43(19%)	0.713

No abnormalities, no changes of CSF WBC count (> 5 cells/μl) and CSF protein concentration (> 45 mg/dl); One abnormality, change in CSF WBC count (> 5 cells/μl) or CSF protein concentration (> 45 mg/dl); Two abnormalities, changes in CSF WBC count (> 5 cells/μl) and CSF protein concentration (> 45 mg/dl); RPR, rapid plasma regain

### Changes of MMSE and RPR titer in serum and CSF

As shown in Tables 3, 13 CSF RPR+ GP patients (including 12 males and 1 female) who received IV penicillin treatment (penicillin G, 24 million units per day for 14 days) had a follow-up evaluation including MMSE and RPR tests in both serum and CSF. The median MMSE change (the changes in MMSE score between last follow-up evaluation in 12 months after penicillin treatment and initial admission) was 4 (IQR 2–11). None of the patients who received the follow-up evaluation showed obvious cognitive deterioration on clinical examination. MMSE scores were higher at post-treatment as compared to pre-treatment (*P* < 0.001, see Table 3). For CSF RPR+ patients, the CSF RPR titer declined four-fold in 85% (11/13) of the patients, whereas the serum RPR titer declined four-fold in only 46% (6/13) of the patients. The odds ratio is 6.4 (95% confidence interval 1.0–41.2). The changes of CSF WBC and protein measures in 12 patients by follow-up are shown in Table 3.

**Table 3:** MMSE and changes of CSF measures and serum RPR titer in CSF RPR+ patients by follow-up

Variable	pre-treatment	12 months after treatment	*P*
Increased CSF WBC count^a^	5/12(42%)	0/12(0%)	0.012
Increased CSF protein concentration^a^	7/12(58%)	2/12(17%)	0.035
Four-fold decline in CSF RPR	–	11/13(85%)	–
Four-fold decline in serum RPR	–	6/13(46%)	–
Median MMSE (IQR)	13(9.5–15)	19(16.5–20.5)	< 0.001

Increased CSF WBC count means CSF WBC count > 5 cells/μl; Increased CSF protein concentration means CSF protein concentration > 45 mg/dl; RPR, rapid plasma regain; MMSE, mini-mental state examination; ^a^Data are for 12 patients

Fourteen CSF RPR- GP patients (including 13 males and 1 female) had a follow-up evaluation of serum RPR titer (see Table 1), while 6 of them had a follow-up evaluation of both CSF and serum RPR titer. The changes of MMSE scores and serum and CSF measures in these 6 patients by follow-up are shown in Table 4.

**Table 4:** MMSE and changes of CSF measures and serum RPR titer in CSF RPR- patients by follow-up

Variable	pre-treatment	12 months after treatment	*P*
Increased CSF WBC count	2/6(33%)	1/6(17%)	0.505
Increased CSF protein concentration	6/6(100%)	1/6(17%)	0.003
Four-fold decline in CSF RPR	–	–	–
Four-fold decline in serum RPR	–	3/6(50%)	–
Median MMSE (IQR)	14.5(11–18.25)	17(13–19.25)	0.048

Increased CSF WBC count means CSF WBC count > 5 cells/μl; Increased CSF protein concentration means CSF protein concentration > 45 mg/dl; RPR, rapid plasma regain; MMSE, mini-mental state examination

## Discussion

In this study, we explored the correlation between the RPR titer and GP. Serum RPR titer ≥1:32 has been described as a risk factor for neurosyphilis with HIV infection regardless of the stage of syphilis [7–9]. In this study, we also found that the number of CSF RPR+ patients with serum RPR titer ≥1:32 was significantly higher when compared with CSF RPR- patients.

When patients have symptomatic neurosyphilis, the success of therapy is assessed by normalization of CSF abnormalities, including pleocytosis, elevated protein concentration, or a reactive CSF VDRL test, and by resolution of symptoms and signs [5]. Repeated determination of serum RPR titer is recommended to evaluate treatment response, with a four-fold decrease from baseline and/or seroreversion in 12–24 months after treatment representing an appropriate response to therapy/serologic cure [4]. Marra et al. found that a four-fold decline in serologic RPR titers correlated with resolution of CSF parameters in persons with neurosyphilis, and normalization of serum RPR titer correctly predicted treatment success of neurosyphilis [10]. However, we found a four-fold decline in CSF RPR titer is more likely to occur than four-fold decline in serum RPR titer among these CSF RPR+ GP patients after penicillin treatment. Reactive non-treponemal test in non-HIV-infected late (latent) syphilis patients often remains stable after adequate therapy [2]. Many studies have found the serological response of nontreponemal tests (ie, VDRL, RPR) to treatment was influenced by syphilis stage but not by HIV infection or reinfection [11–14]. Compared to primary syphilis, later stages of syphilis showed a significantly slower treatment response [11, 13–17]. In this study, all the GP patients were late syphilis, which probably explains the 4-fold decline in serum RPR titers might not adequately predict treatment efficacy in GP patients. The majority of patients were early neurosyphilis in the study by Marra et al. [10], which could partially explain why a four-fold decline in CSF RPR titer instead of the serum RPR normalization found by Marra is a good predictor of treatment efficacy in HIV-negative CSF RPR+ GP patients in our finding. According to these results, it should be emphasized that the disease stage of syphilis might lead to different treatment response. Different recommendations may be needed for the follow-up of neurosyphilis depending on the stage of the disease.

During the follow-up at 12 months after penicillin treatment, no significant difference was found in the four-fold decline in serum RPR titer between CSF RPR+ and CSF RPR- patients. According to our data and our analyses, for CSF RPR- patients, normalization of CSF measures, not serum RPR titer value, is a sensible way of assessing treatment efficacy. A cutoff of greater than or equal to 5 cells/μL has been the standard for determining normal CSF values. For CSF RPR- GP patients, CSF WBC count was often normal before treatment, so it is difficult to evaluate the treatment efficacy by assessing the recovery of CSF WBC. CSF protein concentration in neurosyphilis is slow to normalize and may remain elevated even after the normalization of other CSF and clinical abnormalities [10, 18]. Thus, the decision to re-treat should not be based solely on failure of CSF protein concentration to normalize [1]. Importantly, our study found that the number of CSF RPR- patients (91%) with CSF protein concentration > 45 mg/dl was significantly higher when compared with CSF RPR+ patients (74%) (*P* = 0.028). The normalization of CSF protein concentration occurred in most of CSF RPR- GP patients by follow-up, which suggested that it may be a good indicator of the success of therapy in GP, although the number of this patient group by follow-up is relatively small. Further studies should be conducted to provide more evidence on this question.

Some limitations of our study should be considered. Specifically, a) the study is a retrospective study, in which case the association may be confounded or modified by patient characteristics; b) the large percentage of loss of follow-up leads to small number of the patients with follow-up, which has limited the statistical power; c) further studies are required to assess an association between RPR titers and the clinical features of these cases, and to identify an ideal indicator of the success of therapy for CSF-RPR- GP patients.

## Conclusions

The practical implications of our findings are that a four-fold decline in CSF RPR titer is a good predictor for treatment efficacy in CSF RPR+ GP patients within 12 months after completion of therapy. The sole use of serum RPR titer might not adequately predict treatment efficacy of GP. Considering the limitations of this study, larger samples and more follow-up data are needed for future investigation. Furthermore, the association between RPR titers and the clinical features of these cases need to systematically quantified, and more indicators of treatment efficacy need to be investigated for CSF RPR- GP patients.

## Additional files

Additional file 1: **Table S1.** Follow-up of MMSE, CSF and serum measures in CSF-RPR+ patients. Thirteen CSF RPR+ GP patients returned for follow-up visits with lumbar puncture, MMSE scores and blood samples in 12 months after penicillin treatment. (DOCX 19 kb)

Additional file 2: **Table S2.** Follow-up of MMSE, CSF and serum measures in CSF-RPR- patients. Fourteen CSF RPR- GP patients returned for follow-up visits with MMSE scores and serological tests in 12 months after penicillin treatment, while 6 of them had a follow-up evaluation of CSF. (DOCX 19 kb)

## Abbreviations

CSF: cerebrospinal fluid
GP: general paresis
MMSE: Mini-Mental State Examination
RPR: rapid plasma regain
TPPA: *T. pallidum* Particle Agglutination test
VDRL: Venereal disease research laboratory
WBC: white blood cells
