# Supplementary material for: The performance of rapid plasma reagin (RPR) titer in HIV-negative general paresis after neurosyphilis therapy
Source: BMC Infect Dis. 2018 Apr 2;18:144. doi: 10.1186/s12879-018-3062-4 (PMC5879544; doi:10.1186/s12879-018-3062-4)
Supplement: Supplementary file 1 — Table S1. Follow-up of MMSE, CSF and serum measures in CSF-RPR+ patients. Thirteen CSF RPR+ GP patients returned for follow-up visits with lumbar puncture, MMSE scores and blood samples in 12 months after penicillin treatment. (DOCX 19 kb) [file 12879_2018_3062_MOESM1_ESM.docx]

**Additional file 1**

**Table S1. Follow-up of MMSE, CSF and serum measures in CSF-RPR+ patients.**

|  |  | CSF-RPR+ GP | | | | |
| --- | --- | --- | --- | --- | --- | --- |
|  |  | pre-treatment | 3 months | 6 months | 9 months | 12 months |
| ***1*** | WBC | 0 |  |  | 0 | 0 |
|  | Pr | 0.67 |  |  | 0.25 | 0.30 |
|  | CSF-RPR | 1：2 |  |  | Non-reactive | Non-reactive |
|  | Serum-RPR | 1：8 |  |  | 1：4 | 1：4 |
|  | MMSE | 13 |  |  | 17 | 18 |
| ***2*** | WBC | 5 |  | 12 | 0 | 2 |
|  | Pr | 1.1 |  | 0.74 | 0.55 | 0.52 |
|  | CSF-RPR | 1：2 |  | 1：2 | 1：2 | 1：2 |
|  | Serum-RPR | 1：128 |  | 1：8 | 1：32 | 1：8 |
|  | MMSE | 11 |  | 11 | 13 | 13 |
| ***3*** | WBC | 4 | 2 |  | 2 | 0 |
|  | Pr | 0.4 | 0.35 |  | 0.23 | 0.26 |
|  | CSF-RPR | 1：1 | 1:1 |  | Non-reactive | Non-reactive |
|  | Serum-RPR | 1：32 | 1：32 |  | 1：4 | 1：4 |
|  | MMSE | 15 | 15 |  | 17 | 19 |
| ***4*** | WBC | 4 |  | 2 |  | 0 |
|  | Pr | 0.34 |  | 0.35 |  | 0.26 |
|  | CSF-RPR | 1：2 |  | 1：1 |  | Non-reactive |
|  | Serum-RPR | 1：64 |  | 1：32 |  | 1：4 |
|  | MMSE | 17 |  | 17 |  | 20 |
| ***5*** | WBC | 2 |  |  |  | 0 |
|  | Pr | 0.3 |  |  |  | 0.27 |
|  | CSF-RPR | 1：2 |  |  |  | Non-reactive |
|  | Serum-RPR | 1：32 |  |  |  | 1：4 |
|  | MMSE | 14 |  |  |  | 16 |
| ***6*** | WBC | 0 |  |  | 1 | 0 |
|  | Pr | 0.36 |  |  | 0.28 | 0.33 |
|  | CSF-RPR | 1：2 |  |  | Non-reactive | Non-reactive |
|  | Serum-RPR | 1：8 |  |  | 1：8 | 1：8 |
|  | MMSE | 18 |  |  | 18 | 18 |
| ***7*** | WBC |  |  |  |  |  |
|  | Pr |  |  |  |  |  |
|  | CSF-RPR | 1：2 |  |  |  | Non-reactive |
|  | Serum-RPR | 1：4 |  |  |  | 1：4 |
|  | MMSE | 15 |  |  |  | 22 |
| ***8*** | WBC | 10 |  |  |  | 4 |
|  | Pr | 0.69 |  |  |  | 0.37 |
|  | CSF-RPR | 1：8 |  |  |  | 1：4 |
|  | Serum-RPR | 1：32 |  |  |  | 1：32 |
|  | MMSE | 13 |  |  |  | 13 |
| ***9*** | WBC | 16 |  |  | 0 | 0 |
|  | Pr | 0.61 |  |  | 0.53 | 0.49 |
|  | CSF-RPR | 1：4 |  |  | Non-reactive | Non-reactive |
|  | Serum-RPR | 1：16 |  |  | 1：8 | 1：8 |
|  | MMSE | 15 |  |  | 17 | 17 |
| ***10*** | WBC | 9 |  |  |  | 0 |
|  | Pr | 0.42 |  |  |  | 0.25 |
|  | CSF-RPR | 1：2 |  |  |  | Non-reactive |
|  | Serum-RPR | 1：16 |  |  |  | 1：8 |
|  | MMSE | 12 |  |  |  | 20 |
| ***11*** | WBC | 49 |  |  | 2 | 3 |
|  | Pr | 0.63 |  |  | 0.35 | 0.37 |
|  | CSF-RPR | 1：2 |  |  | 1：2 | Non-reactive |
|  | Serum-RPR | 1：8 |  |  | 1：4 | 1：2 |
|  | MMSE | 5 |  |  | 16 | 20 |
| ***12*** | WBC | 20 |  |  | 0 | 2 |
|  | Pr | 0.82 |  |  | 0.37 | 0.41 |
|  | CSF-RPR | 1：2 |  |  | Non-reactive | Non-reactive |
|  | Serum-RPR | 1：64 |  |  | 1：4 | 1：1 |
|  | MMSE | 6 |  |  | 20 | 25 |
| ***13*** | WBC | 0 |  |  |  | 0 |
|  | Pr | 0.62 |  |  |  | 0.44 |
|  | CSF-RPR | 1：4 |  |  |  | 1：1 |
|  | Serum-RPR | 1：32 |  |  |  | 1：32 |
|  | MMSE | 7 |  |  |  | 21 |
