# Supplementary material for: The performance of rapid plasma reagin (RPR) titer in HIV-negative general paresis after neurosyphilis therapy
Source: BMC Infect Dis. 2018 Apr 2;18:144. doi: 10.1186/s12879-018-3062-4 (PMC5879544; doi:10.1186/s12879-018-3062-4)
Supplement: Supplementary file 2 — Table S2. Follow-up of MMSE, CSF and serum measures in CSF-RPR- patients. Fourteen CSF RPR- GP patients returned for follow-up visits with MMSE scores and serological tests in 12 months after penicillin treatment, while 6 of them had a follow-up evaluation of CSF. (DOCX 19 kb) [file 12879_2018_3062_MOESM2_ESM.docx]

**Additional file 2**

**Table S2. Follow-up of MMSE, CSF and serum measures in CSF-RPR- patients.**

|  |  | CSF-RPR- GP | | | | |
| --- | --- | --- | --- | --- | --- | --- |
|  |  | pre-treatment | 3 months | 6 months | 9 months | 12 months |
| ***1*** | WBC | 15 |  |  |  |  |
|  | Pr | 0.76 |  |  |  |  |
|  | CSF-RPR | Non-reactive |  |  |  |  |
|  | Serum-RPR | 1：16 |  | 1：4 |  |  |
|  | MMSE | 13 |  | 15 |  |  |
| ***2*** | WBC | 2 |  |  |  |  |
|  | Pr | 0.72 |  |  |  |  |
|  | CSF-RPR | Non-reactive |  |  |  |  |
|  | Serum-RPR | 1：2 | 1：1 | Non-reactive |  |  |
|  | MMSE | 7 | 10 | 10 |  |  |
| ***3*** | WBC | 6 |  | 2 |  |  |
|  | Pr | 0.84 |  | 0.31 |  |  |
|  | CSF-RPR | Non-reactive |  | Non-reactive |  |  |
|  | Serum-RPR | 1：4 | 1：2 | 1：1 |  |  |
|  | MMSE | 12 | 14 | 15 |  |  |
| ***4*** | WBC | 8 |  |  |  |  |
|  | Pr | 0.38 |  |  |  |  |
|  | CSF-RPR | Non-reactive |  |  |  |  |
|  | Serum-RPR | 1：2 |  |  |  | 1：4 |
|  | MMSE | 20 |  |  |  | 18 |
| ***5*** | WBC | 50 |  | 2 |  | 0 |
|  | Pr | 0.59 |  | 0.28 |  | 0.30 |
|  | CSF-RPR | Non-reactive |  | Non-reactive |  | Non-reactive |
|  | Serum-RPR | 1：4 |  | 1：2 |  | 1：2 |
|  | MMSE | 11 |  | 12 |  | 13 |
| ***6*** | WBC | 2 |  |  |  |  |
|  | Pr | 0.75 |  |  |  |  |
|  | CSF-RPR | Non-reactive |  |  |  |  |
|  | Serum-RPR | 1：16 | 1：4 |  |  |  |
|  | MMSE | 15 | 15 |  |  |  |
| ***7*** | WBC | 2 |  | 2 |  | 6 |
|  | Pr | 0.55 |  | 0.35 |  | 0.3 |
|  | CSF-RPR | Non-reactive | Non-reactive | Non-reactive |  | Non-reactive |
|  | Serum-RPR | 1：8 | 1：8 | 1：4 |  | 1：4 |
|  | MMSE | 19 | 19 | 19 |  | 19 |
| ***8*** | WBC | 3 |  |  | 8 |  |
|  | Pr | 0.51 |  |  | 0.57 |  |
|  | CSF-RPR | Non-reactive |  |  | Non-reactive |  |
|  | Serum-RPR | 1：8 |  | 1：4 | Non-reactive |  |
|  | MMSE | 18 |  | 18 | 20 |  |
| ***9*** | WBC | 0 |  |  | 0 | 0 |
|  | Pr | 0.99 |  |  | 0.44 | 0.40 |
|  | CSF-RPR | Non-reactive |  |  | Non-reactive | Non-reactive |
|  | Serum-RPR | 1：16 | 1：16 |  | 1：8 | 1:8 |
|  | MMSE | 17 | 17 |  | 17 | 19 |
| ***10*** | WBC | 2 | 2 |  | 4 |  |
|  | Pr | 0.47 | 0.37 |  | 0.28 |  |
|  | CSF-RPR | Non-reactive | Non-reactive |  | Non-reactive |  |
|  | Serum-RPR | 1：32 | 1：8 |  | 1：8 |  |
|  | MMSE | 11 | 12 |  | 13 |  |
| ***11*** | WBC | 3 |  |  |  |  |
|  | Pr | 0.69 |  |  |  |  |
|  | CSF-RPR | Non-reactive |  |  |  |  |
|  | Serum-RPR | 1：2 |  | 1：2 |  | 1:2 |
|  | MMSE | 18 |  | 18 |  | 20 |
| ***12*** | WBC | 2 |  |  |  |  |
|  | Pr | 0.59 |  |  |  |  |
|  | CSF-RPR | Non-reactive |  |  |  |  |
|  | Serum-RPR | 1：8 |  | Non-reactive |  |  |
|  | MMSE | 12 |  | 15 |  |  |
| ***13*** | WBC | 8 |  |  |  |  |
|  | Pr | 0.55 |  |  |  |  |
|  | CSF-RPR | Non-reactive |  |  |  |  |
|  | Serum-RPR | 1：4 | 1：8 | 1：2 |  | 1：1 |
|  | MMSE | 15 | 15 | 17 |  | 13 |
| ***14*** | WBC | 0 |  |  |  |  |
|  | Pr | 0.48 |  |  |  |  |
|  | CSF-RPR | Non-reactive |  |  |  |  |
|  | Serum-RPR | 1：8 |  |  |  | 1：4 |
|  | MMSE | 13 |  |  |  | 13 |

**Note.** The red number presents this patient has follow-up visit of serum RPR titer and CSF examination simultaneously.
